# Physical Characteristics, Blue-Green Band Emission and Photocatalytic Activity of Au-Decorated ZnO Quantum Dots-Based Thick Films Prepared Using the Doctor Blade Technique

**DOI:** 10.3390/molecules28124644

**Published:** 2023-06-08

**Authors:** Amanullah Fatehmulla, Belqes A. Shamsan, Ahmed M. El-Naggar, Abdullah M. Aldhafiri, Nilam Qureshi, Taesung Kim, Muhammad Atif, Asif Mahmood, Mohammad Asif

**Affiliations:** 1 Department of Physics and Astronomy, College of Science, King Saud University, Riyadh 11451, Saudi Arabia; 2 Nano Particles Technology Laboratory, School of Mechanical Engineering, Sungkyunkwan University, Suwon 16419, Gyeonggi-do, Republic of Korea; 3 Department of Chemical Engineering, College of Engineering, King Saud University, Riyadh 11421, Saudi Arabia

**Keywords:** co-precipitation, ZnO quantum dots, Au nanoparticles, blue-green band emission, photocatalysis, photoactive

## Abstract

Nanoscale ZnO is a vital semiconductor material whose versatility can be enhanced by sensitizing it with metals, especially noble metals, such as gold (Au). ZnO quantum dots were prepared via a simple co-precipitation technique using 2-methoxy ethanol as the solvent and KOH as the pH regulator for hydrolysis. The synthesized ZnO quantum dots were deposited onto glass slides using a simple doctor blade technique. Subsequently, the films were decorated with gold nanoparticles of different sizes using a drop-casting method. The resultant films were characterized via various strategies to obtain structural, optical, morphological, and particle size information. The X-ray diffraction (XRD) reveals the formation of the hexagonal crystal structure of ZnO. Upon Au nanoparticles loading, peaks due to gold are also observed. The optical properties study shows a slight change in the band gap due to Au loading. Nanoscale sizes of particles have been confirmed through electron microscope studies. P.L. studies display blue and blue-green band emissions. The significant degradation efficiency of 90.2% methylene blue (M.B.) was attained in natural pH in 120 min using pure ZnO catalyst while one drop gold-loaded catalysts, ZnO: Au 5 nm, ZnO: Au 7 nm, ZnO: Au 10 nm and ZnO: Au 15 nm, delivered M.B. degradation efficiency of 74.5% (in 245 min), 63.8% (240 min), 49.6% (240 min) and 34.0% (170 min) in natural pH, respectively. Such films can be helpful in conventional catalysis, photocatalysis, gas sensing, biosensing, and photoactive applications.

## 1. Introduction

For many II-VI and III-V compounds, synthetic paths have been established which produce monodisperse colloidal nanocrystals approximately with a well-controlled size in the 1–10 range. These insulating nanocrystals exhibit discrete atomic-like valence (hole) and conduction (electron) levels owing to size confinement; the energy level spectrum can be regulated within certain limits by varying the size of the dots. Therefore, they are termed as artificial atoms or quantum dots [1]. Zinc oxide (ZnO) heterostructures and nanostructures have been used as transparent conductors in solar cells, components in high-power electronics, gas, chemical sensors, and U.V. light emitters [2]. Among these compounds, ZnO, an intrinsically n-type material, has a very large exciton binding energy (60 meV) compared to other materials with a wide bandgap [3], which leads to more efficient excitonic emission at room temperature. In addition, the room temperature ferromagnetic ordering in ZnO can be mediated by the exchange interaction between spins of acceptor-bound charge carriers [4]. Lately, ZnO nanostructures have grabbed particular focus for possible applications in spintronic and optoelectronic devices, such as emitting and laser diodes with polarized output, spin-based memory, and logic [5,6]. ZnO-based nanostructures have garnered attention because of their excellent properties and potential applications in the ultraviolet to violet light-emitting, spintronic, and other optoelectronic devices [7]. In ZnO nanostructures, one can enhance the needed properties, such as considerable exciton binding energy 60 meV [8], while eliminating unwanted properties of bulk ZnO, such as weak exciton emission compared with the defect-related deep-level visible emission. The large exciton binding energy and strong exciton emission would permit stable high-yield luminescence from ZnO nanostructures even at room temperature [9,10].

Global warming and environmental pollution are the foremost worries due to heavy metal pollution. These heavy metals and their associated compounds are damaging and unsafe for human health. As such, the degradation of such pollutants has become the need of the hour. Photocatalysis quickly degrades the organic pollutants and converts them into harmless and nonhazardous products [11,12]. Due to rising urbanization and industrialization, water adulteration has developed as a global problem in water refinement. Removing emerging pollutants, such as dyes and antibiotics, is unusual for conventional water treatment plants. Methylene blue (M.B.) and oxytetracycline (OTC) were degraded using Fenton-activated catalysts under dark as well as visible light conditions [13]. Several approaches, such as the Fenton method, electrochemical process, ozonation, and adsorption, are used to treat industrial wastewater. However, the photocatalysis treatment method is ideal because upon irradiation of visible light on porous semiconductor material, hydroxyl radical is formed, a potent oxidizing agent used to degrade objective dye [14]. Up to now, reports on the production of MOFs-derived LDHs are intermittent; it is crucial to understand the fabrication of MOFs-based materials for environmental applications [15]. Many transition metal oxide catalysts were blended and engaged for dye degradation drives using direct sunlight or U.V. light [16,17]. Among all transition metal photocatalysts, zinc oxide photocatalysts are more efficient in photocatalytic depolymerization and in minimizing environmental problems [18]. Zinc oxide (ZnO) is a valuable material with possible applications in photocatalysts, solar cells, gas sensors, varistors, piezoelectric transducers, transparent electrodes, electroluminescent devices, and ultraviolet laser diodes. As a result, it has stimulated broad research [19,20].

ZnO nanostructure’s structural, morphological, optical, and electronic properties can be enriched by doping/sensitizing/loading/decorating it with different materials, especially noble metals [21,22,23,24]. Au is one of the significant noble metals and does not undergo rustiness in photocatalytic processes, and therefore is an important material to be used with ZnO [25]. Au/ZnO composites have unique physical and chemical properties, such as surface electron transfers, more oxygen vacancies microstructures, and the chemical and electronic sensitization of Au nanoparticles [26,27,28,29,30,31]. In this context, studies on sensitizing with a noble metal, such as Au nanoparticles (Au NPs) of various sizes loaded on the nanoscale ZnO-based thick film surfaces to enhance their photo-activity, are presented in this paper.

## 2. Results and Discussion

### 2.1. X-ray Diffraction Study

**Figure 1:**
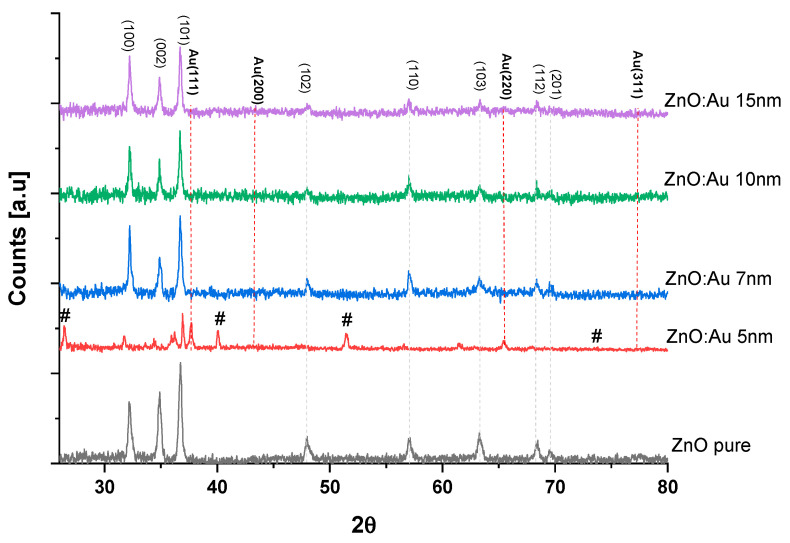
XRD patterns for pure ZnO and one drop Au with different sizes: (**a**) ZnO: Au 5 nm (#: HAuCl4), (**b**) ZnO: Au 7 nm, (**c**) ZnO: Au 10 nm, (**d**) ZnO: Au 15 nm.

X-ray diffractograms of the prepared pure and Au nanoparticles-decorated ZnO thick films are shown in Figure 1. The obtained diffraction patterns match the standard ZnO diffraction pattern corresponding to JCPDS card no. 36-1451. This XRD pattern for undoped ZnO thick films shows the diffraction peaks for the planes (100), (002), (101), (102), (110), (103), (112), and (201) with corresponding 2θ values of 31.77°, 34.42°, 36.25°, 47.53°, 56.60°, 62.86°, 67.96°, and 69.1°, respectively, as shown in Figure 1. These diffraction peaks can be attributed to crystalline ZnO with a hexagonal crystal structure. For gold-decorated ZnO thick films, additional low-intensity peaks due to Au decoration of planes (111), (200), (220), and (311) corresponding to 2θ = 38.25, 44.46, 64.69, and 77.72 are also observed. The XRD results are consistent with available reports on Au–ZnO nanostructures [26]. Most of the films have the most substantial reflection at (100), (002), and (101) planes, which indicates the preferential orientation along the c-axis for all samples [18,32].

The average crystallite size D was calculated using the Debye–Scherrer equation [33]:

D = (k λ)/(β cos θ)(1)

where, the constant k equals 0.9, the wavelength of the X-ray (λ = 1.5405 Å), β is the full width at half maximum (FWHM), and θ is the diffraction angle. We notice that the crystallite size increases with the gradual increase in gold nanoparticle size in the solution used for drop-casting. It is reported that the grain size of the Au/ZnO thin film increased when the thin film contained a thicker AuNP layer [34,35]. In the available reports, on the Au NPs which were sputtered onto the ZnO thin film, lattice expansion occurred because of the higher ionic radius of Au3+ (0.85 Å) compared with that of Zn2+ (0.74 Å), which resulted in the expansion of the lattice bond length and an increase in the grain size of the Au-sputtered ZnO thin films. The lattice strain of the undoped ZnO and Au-doped films was calculated using the Stokes–Wilson equation [36]. Their results indicated that the Au-sputtered ZnO nanocomposites prepared with different thicknesses of Au layers (10–50 nm) exhibited changes in their lattice behavior [34,35]. These results match our observations.

Ɛ = β cosθ/4(2)

**Figure 2:**
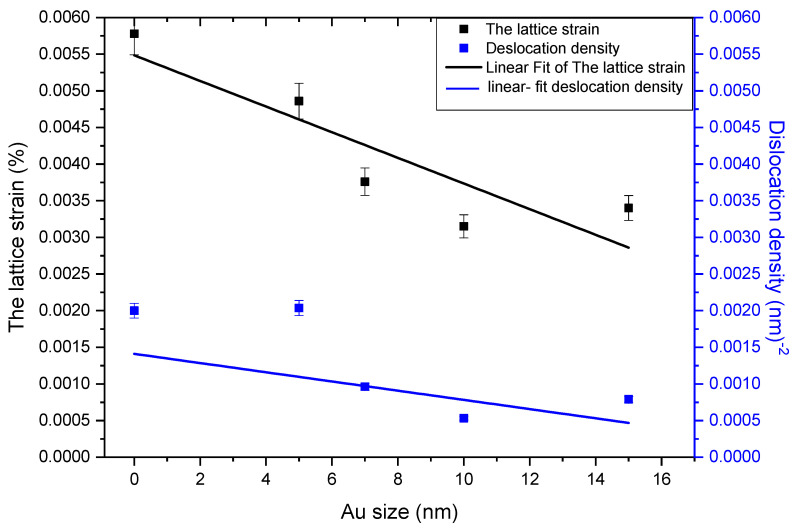
Average lattice strain and dislocation density of pure and ZnO:−Au thick films calculated from XRD data.

From Figure 1, we notice that the FWHM of the (002) peak narrows as the Au dopant increases (in contrast with the report by Davide Stefani [37] and the location of the peak (2θ) and shifts to slightly lesser values compared to the pure ZnO. It showed a decreasing trend (Figure 2). The trend of lattice strain (є) obtained from the XRD data of the peak corresponding to the (002) plane is shown in Figure 1. These changes are ascribed to different grain sizes (low intensity, and peak narrowing) and inner compressive strain (shift of the peak) [38,39]. The number of defects in the sample represents dislocation density (ρ), which is defined as the length of dislocation lines per unit volume of the crystal and is calculated using the equation (3) given by Aydogu and Sendil [39], as well as the simple approach of Williamson and Smallman [40].

ρ = 1/D^2^ (3)

where, D is the crystallite size. Average dislocation density for pure and ZnO: Au thick films is given in Figure 2. As seen in Figure 2, dislocation density (ρ) showed a decreasing trend up to the films where gold nanoparticles of sizes up to 10 nm are used. A marginal increased value is observed for the ZnO: Au thick film with a 15 nm size gold solution. However, the linear fitting shows a decreasing trend with an acceptable standard error.

### 2.2. FESEM and EDS

**Figure 3:**
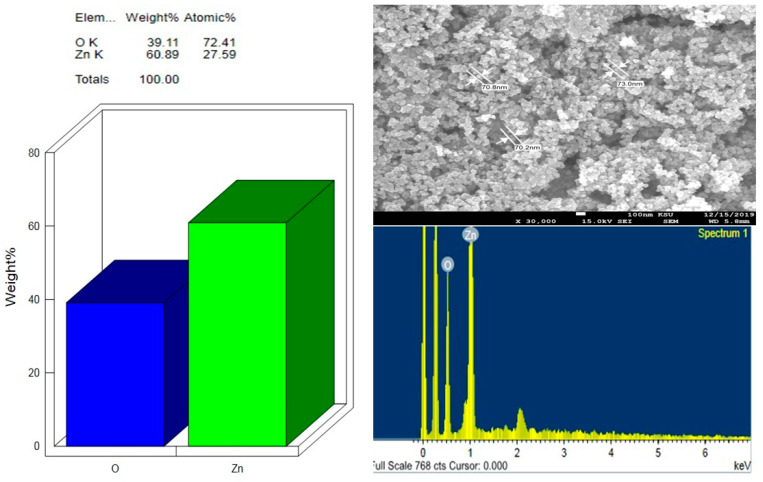
FESEM image of pure ZnO and elemental mapping using EDS.

**Figure 4:**
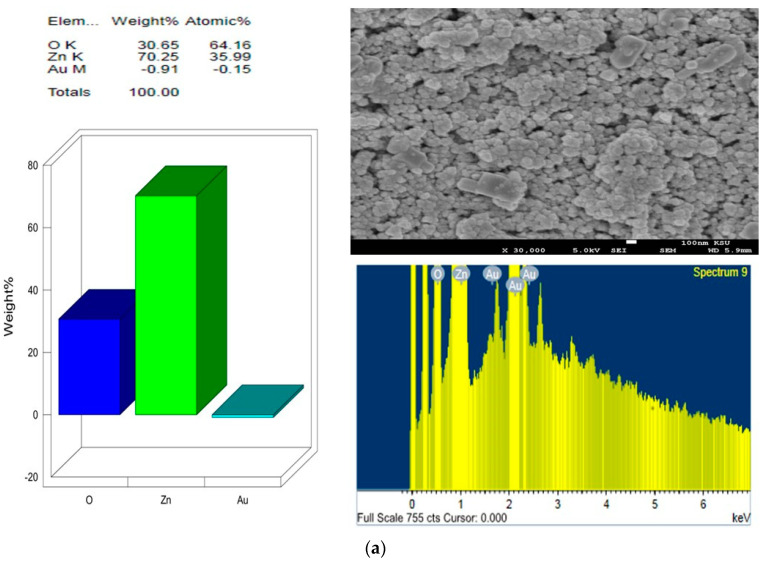

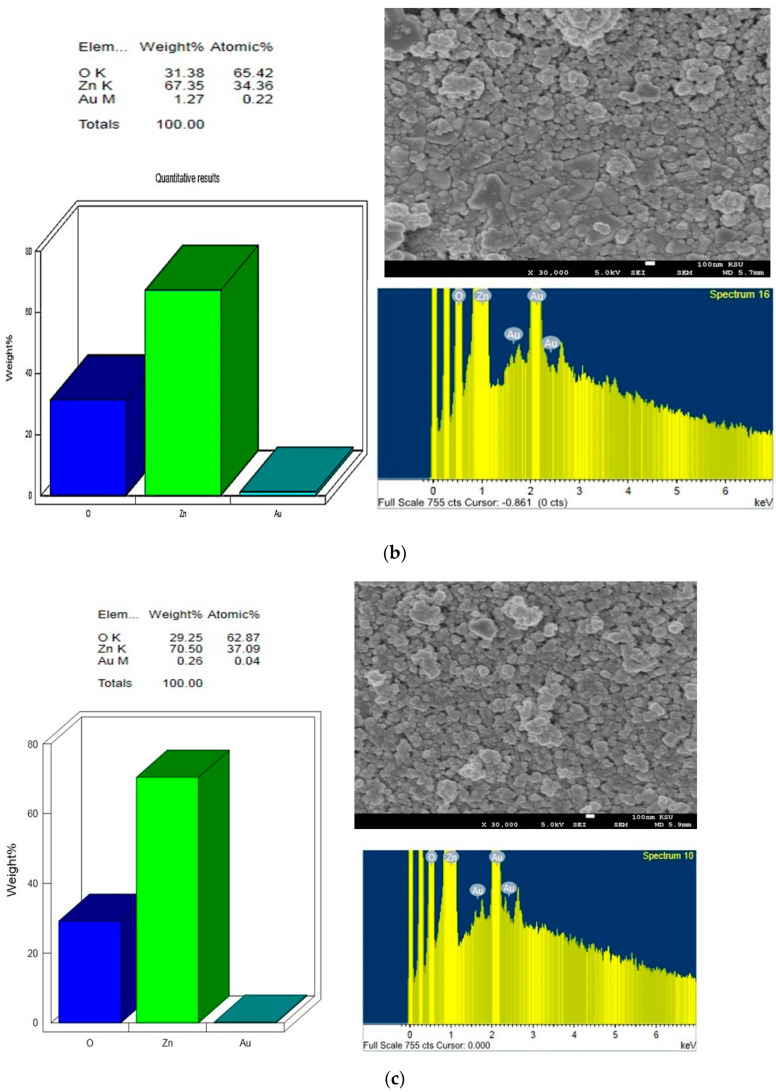

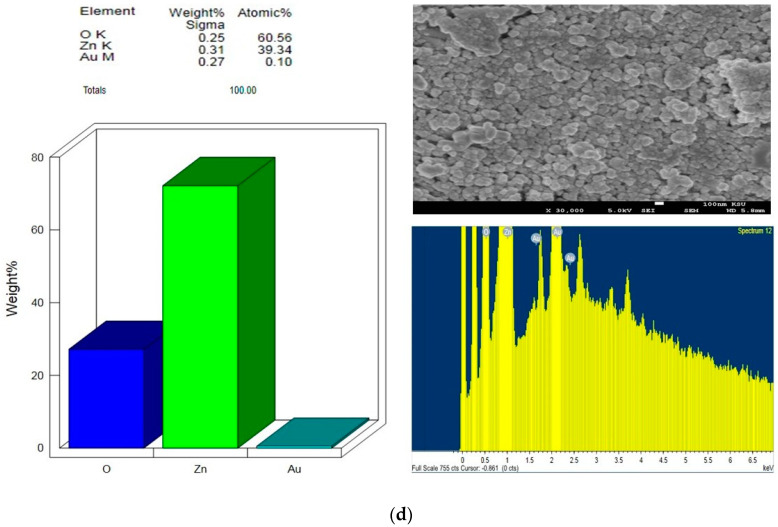
FESEM images of one drop Au-casted films and elemental mapping using EDS: (**a**) ZnO:−Au 5 nm (**b**) ZnO:−Au 7 nm (**c**) ZnO:−Au 10 nm (**d**) ZnO:−Au 15 nm.

Surface and compositional analysis of pure and Au-casted ZnO films were investigated using a 100 nm scale with 30,000 times magnification, as shown in Figure 2, Figure 3 and Figure 4, and Table 1 and Table 2, respectively.

**Figure 5:**
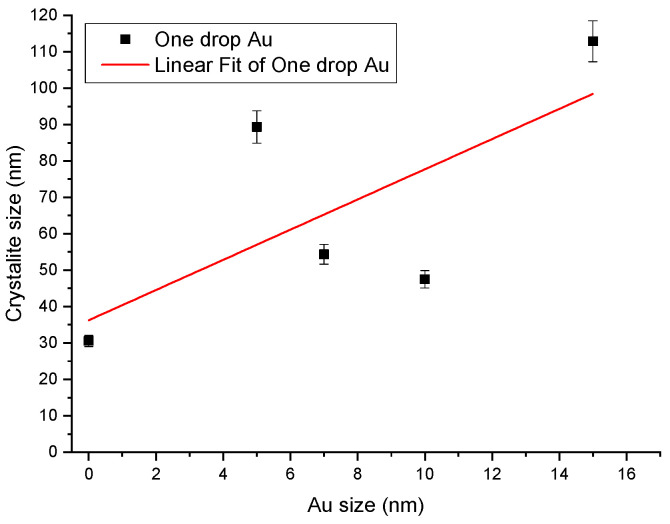
The average crystallite from XRD.

The FESEM images in the figures show that the films’ surface morphology strongly depends on the dopant’s concentration (sizes). It can be seen that the particles are primarily spherical agglomerated cluster shapes and sizes in undoped ZnO (Figure 3). The crystallite size from FESEM images was compared with the calculated average crystallite size obtained using the Debye–Scherrer equation in Table 1. A small amount of gold was detected using EDS in ZnO: Au films with 7, 10, and 15 nm (Figure 4b–d). ZnO-Au, 5 nm sample, displays the occurrence of some large clusters of about 50–100 nm in size, resulting from crystallites aggregation of Au NPs and are visible in these images (Figure 4a), probably because of their low contrast difference as compared to the oxide matrix [41]. The high contrast difference between ZnO and Au is distinguishable due to the higher electron density of the metal Au [42,43]. However, we noticed fewer white particulate patches among the particles in the present case, and such identifications could not be marked. Figure 5 shows the average crystallite size of pure and ZnO: Au films obtained from XRD. The figure demonstrates the linear fit and highlights the error bars.

### 2.3. TEM Images

**Figure 6:**
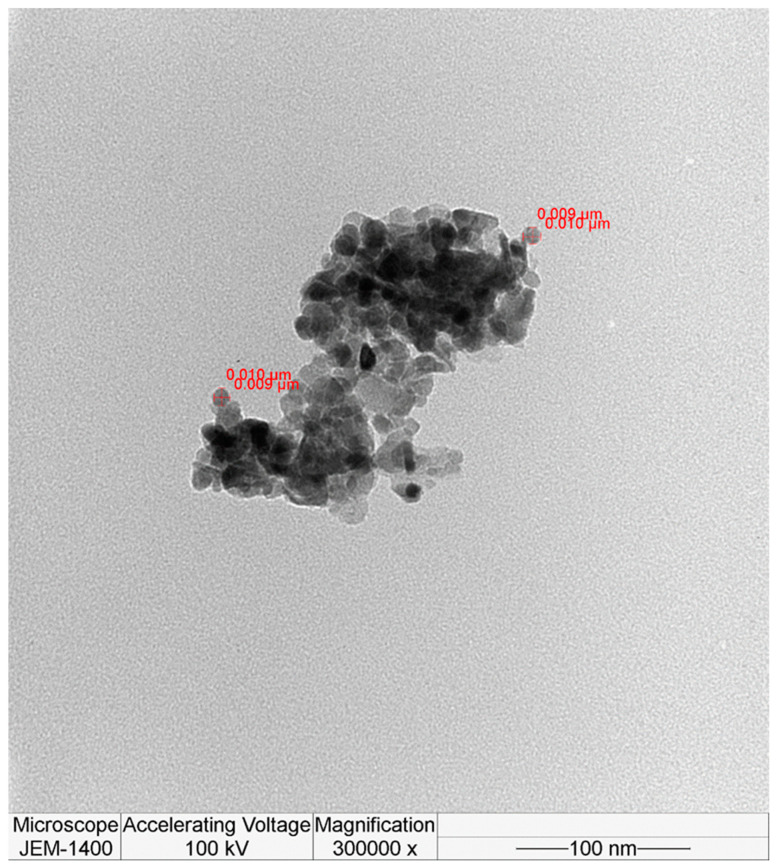
HRTEM image for pure ZnO.

**Figure 7:**
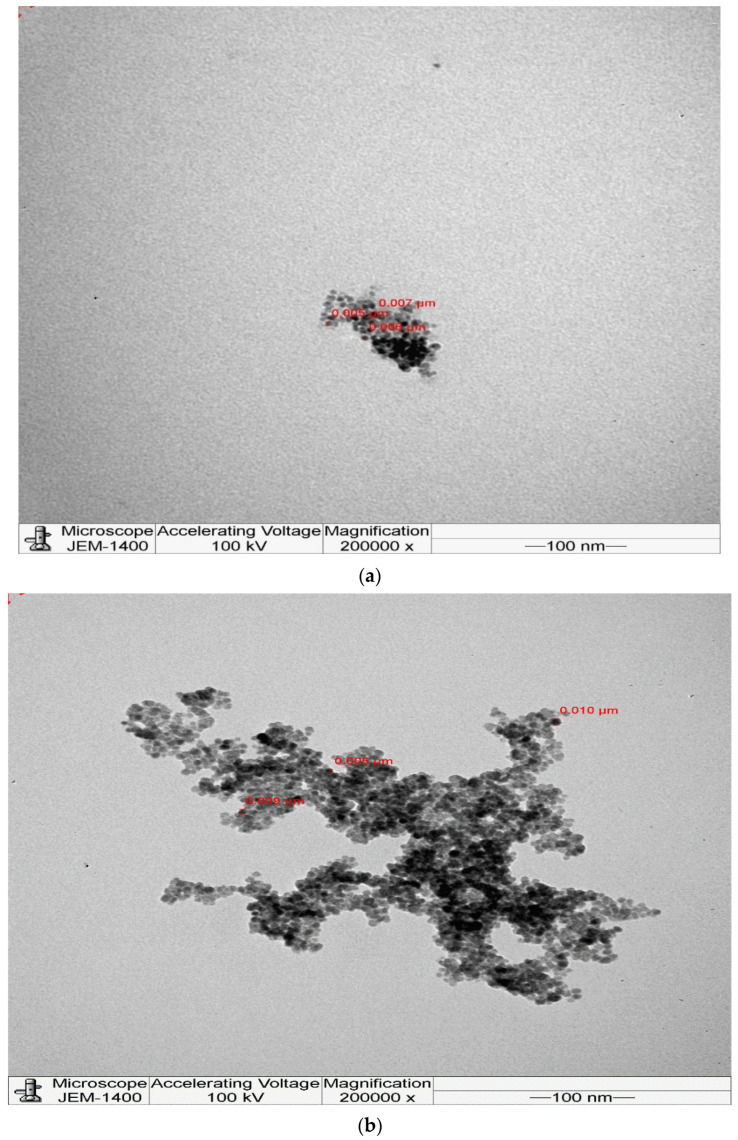

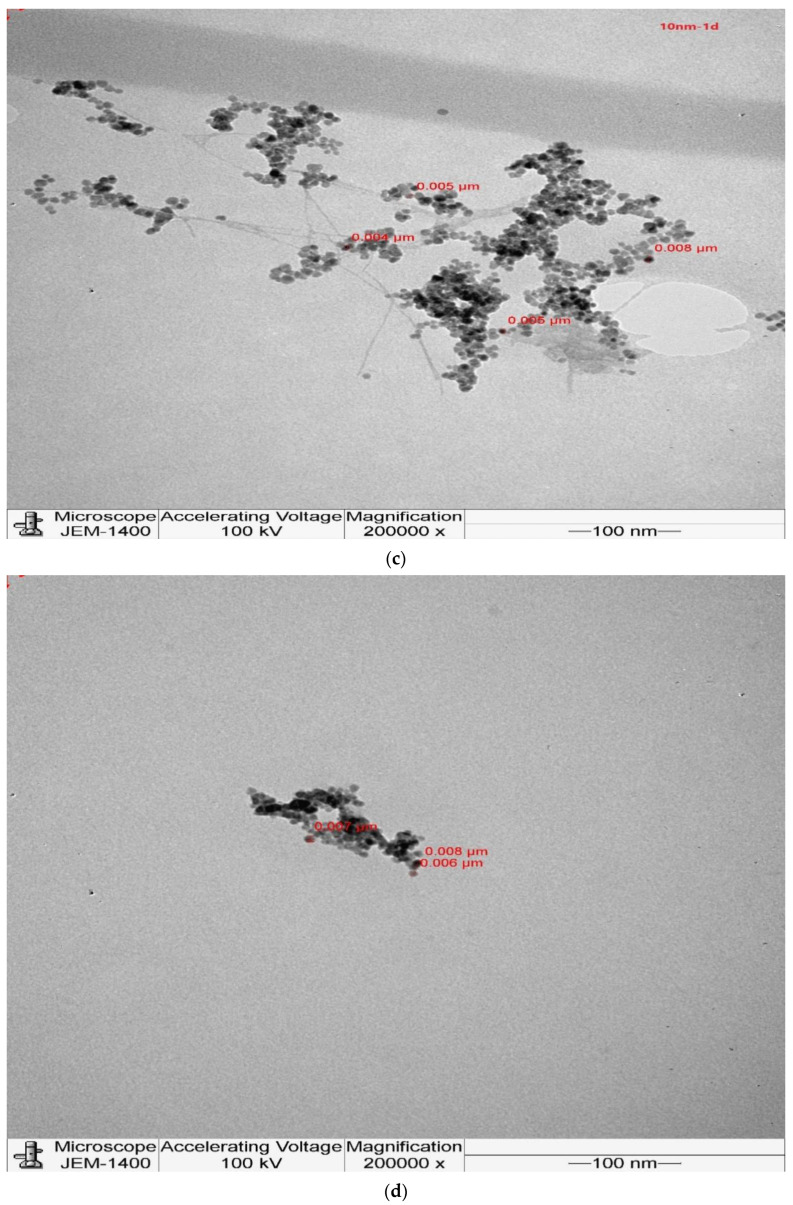
Different HRTEM images for (one drop Au): (**a**) ZnO:−Au 5 nm, (**b**) ZnO:−Au 7 nm, (**c**) ZnO:−Au 10 nm (**d**) ZnO:−Au 15 nm.

The particle size of the pure and Au-decorated thick film samples was determined using TEM. TEM images of pristine and Au-decorated ZnO nanoparticles are shown in Figure 6 and Figure 7, respectively. The HRTEM image of pure ZnO nanoparticles shows the formation of agglomerated nanoparticles of around 10 nm.

HRTEM images of ZnO nanoparticles decorated with Au nanoparticles of different sizes show the formation of nanoparticles (Figure 7a–d) with particle sizes in the 5–10 nm range. However, some particles show a very dark atomic contrast, which is likely attributed to the presence of gold nanoparticles due to their higher atomic number. (Table 2).

The comparison between the XRD, FESEM, and HRTEM results for the crystallite size variation is shown in Table 1 and Table 3, and we note that there is little similarity between XRD and FESEM values. This variation is because FESEM images usually correspond to the grains, whereas XRD analysis gives crystallite size of a particular plane in polycrystalline samples. Table 3 also shows the compositional data obtained from EDS.

### 2.4. UV-Visible Absorption and Transmission Spectra

**Figure 8:**
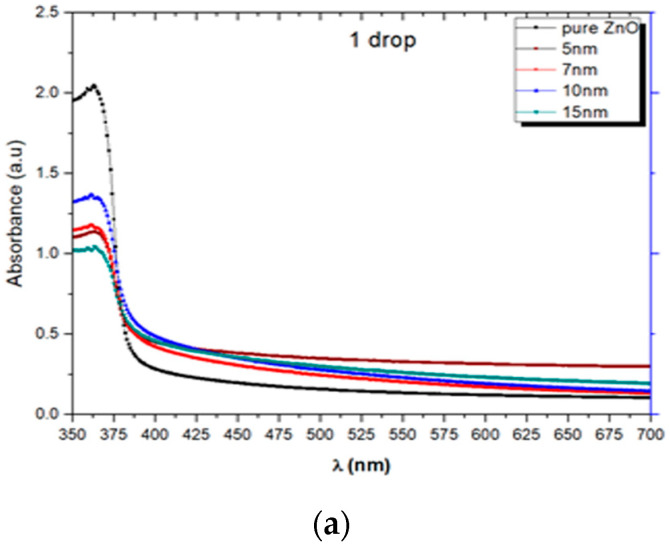

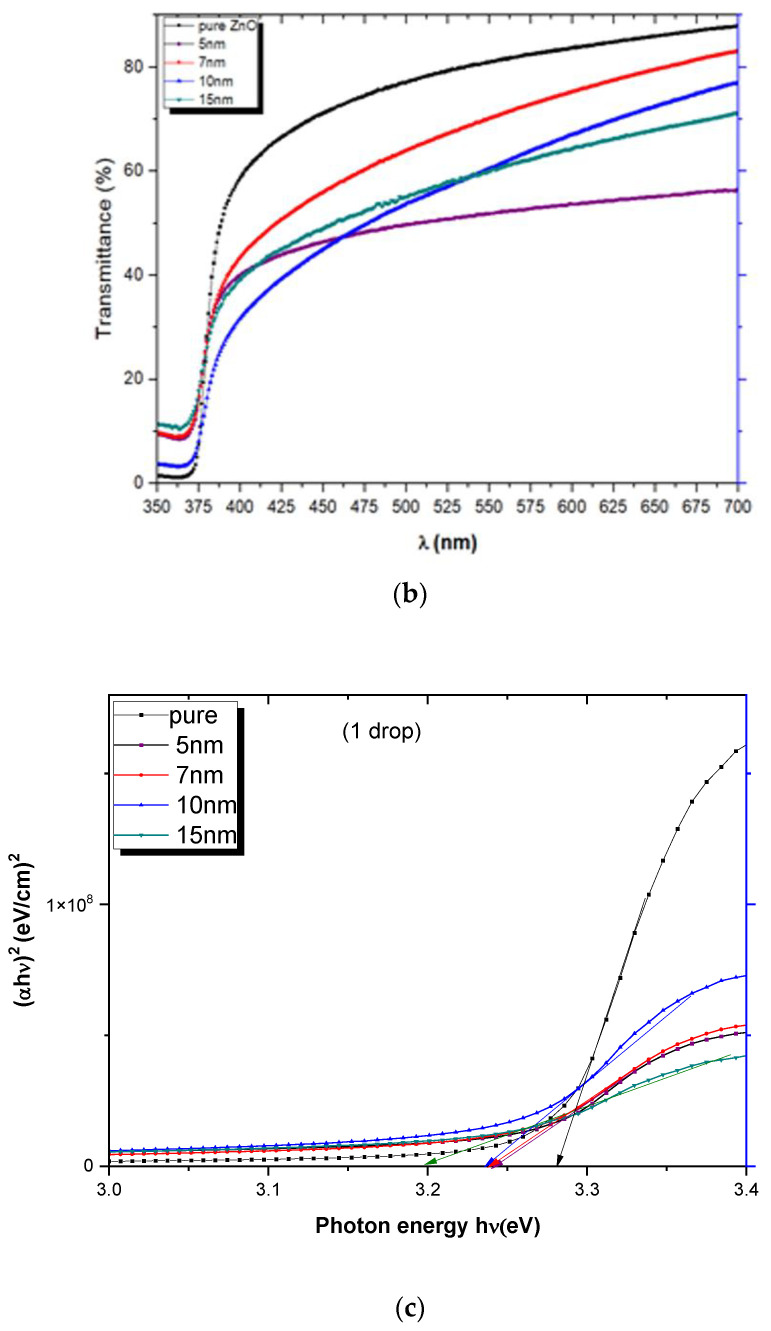
(**a**) Absorption, (**b**) transmission spectra, and (**c**) Tauc plot for pure and Au-decorated ZnO thick films.

The optical properties of pure and Au-decorated ZnO thick films were determined from the absorption and transmission spectra in the 350–700 nm wavelength range and are presented in Figure 8a and Figure 8b, respectively.

Figure 8a shows that the absorption peak for pure ZnO is centered around 365 nm. The absorbance value decreased with Au decoration, and the fundamental absorption band edge is located in the ultraviolet region between 379 and 389 nm. This band edge shifts towards the higher wavelengths (redshift), which is well reported [44].

Figure 8b shows the transmittance spectra for pure and Au-decorated ZnO thick films in the wavelength range between 350 and 700 nm. Pure ZnO film indicates high transmission in the visible region and the absorption edge around 379–389 nm. However, the Au nanoparticles used to decorate ZnO thick films absorb light strongly in the visible part compared to pure ZnO. Due to this, the decrease in transmittance with an increased particle size of Au NPs used for decorating ZnO nanoparticles in the films is observed [45,46]. We observed transmittance between 80% and 30% in the visible region. The reduction in transmittance is attributed to strong scattering and absorption processes. The strong scattering detected is due to grain boundaries; the point defects and disorders in ZnO films occurred due to Au NPs decoration [46]. As shown in Figure 8b, it is observed that the transmittance showed decreasing tendency with Au doping. The reduction in transmittance percentage is because of the progression of Au in the film that enlarges the particle size and slightly contributes to this phenomenon. Moreover, such low transmittance is observed not only because of the increased size of Au NPs but the thin film quality also plays a significant role because of light scattering by grain boundaries [46].

### 2.5. The Band Gap and Tauc’s Plot

There is a direct connection between the U.V. absorption edge and the optical bandgap. In many optical applications, the optical band gap is a significant parameter for Au-decorated thick films. ZnO is considered a direct bandgap semiconductor, and the bandgap can be calculated from the absorption edge using Tauc’s relationship [47]. The optical band gap (Eg) for a direct transition between valence and conduction bands is obtained using the expression (4):

αhν = A (hν − Eg)^n^ (4)

where, (n) equals 1/2, since the transition in ZnO is direct. Hence,

(αhν)^2^ = A (hν − Eg) (5)

where, ‘h’ is Planck’s constant, hν is the photon energy, ‘A’ denotes an energy-independent constant, α is the absorption coefficient of the film, and Eg is the bandgap energy. Eg was obtained by extrapolating the linear portion of Tauc’s plot between (αhν)^2^ and (hν). The Tauc’s plots are shown in Figure 8c.

Figure 8c shows the relation between (αhν)^2^ and (hν), and it represents the energy bandgap of the pure ZnO and ZnO: Au Nps-based thick films, and Table 4 shows the corresponding energy bandgap values. The estimated value for pure ZnO is 3.28 eV, slightly smaller than the bandgap value of 3.34 eV for bulk ZnO [44]. There is a slight difference between the bandgap of pure and the ZnO: Au-decorated ZnO thick films, which submits that the Au doping would lower the bandgap. It is assumed that when an Au atom occupies a Zn site, a robust p–d coupling between Au and O happens that moves the O 2p level up and narrows the direct fundamental bandgap. Thus, the ZnO: Au bandgap is smaller than the ZnO [37].

### 2.6. Photoluminescence Studies (PL)

**Figure 9:**
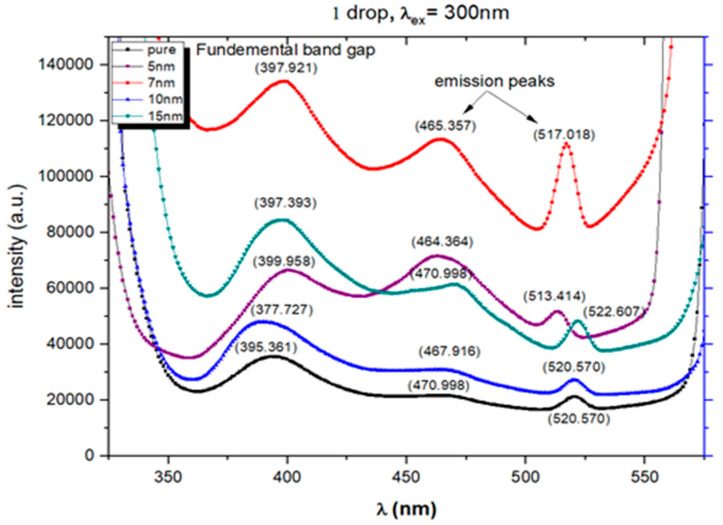
The emission spectra of undoped and ZnO: Au thick films excited at 300 nm.

PL studies are significant in investigating Au-decorated ZnO nanostructures as they are expected to deliver different optoelectronic properties than pure ZnO. The emission spectra were obtained using the spectrofluorophotometer for the pure and Au-decorated ZnO thick films (Figure 9). The excitation wavelength was 300 nm, and the wavelength range for the emission spectra was between 320 and 580 nm, as illustrated in Figure 9.

Figure 9 shows the PL spectra of ZnO and ZnO: Au. We can see that the UV peak is centered around 388 nm for all the films attributed to the exciton peak. The intensities of the peaks in the U.V. region show an increasing trend for all the Au-decorated ZnO films of sizes 5 nm, 7 nm, and 10 nm. There is a probable reason for enhancing UV emission intensity in the ZnO: Au system. The adsorption of visible light by Au forms exciton–surface plasmon coupling that assists in effective electron transfer from Au to ZnO, enhancing UV emission intensity [45,48,49,50,51]. This observation corroborates our results. In another report, the number of single ionized oxygen vacancies (Vo+) are decreased/quenched by catching electrons. When more electrons are transferred from Au to ZnO, a decrease in visible emission intensity has been previously reported, which has not been observed in the present case [51]. In the visible region, some emission peaks are centered around 470 nm and 521 nm, indicating the blue and blue-green bands for all films. Blue band emission is ascribed to the surface defects in ZnO, such as oxygen vacancies and zinc interstitials. Green emission is due to the intrinsic defects of zinc vacancies and oxygen interstitials [52,53].

### 2.7. Photocatalytic Dye Degradation Studies

**Figure 10:**
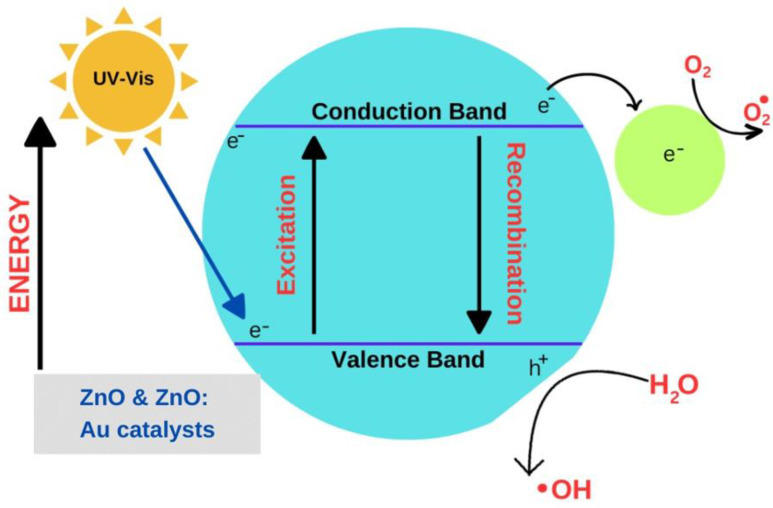
Photocatalytic mechanism of ZnO and ZnO:−Au catalysts.

**Figure 11:**
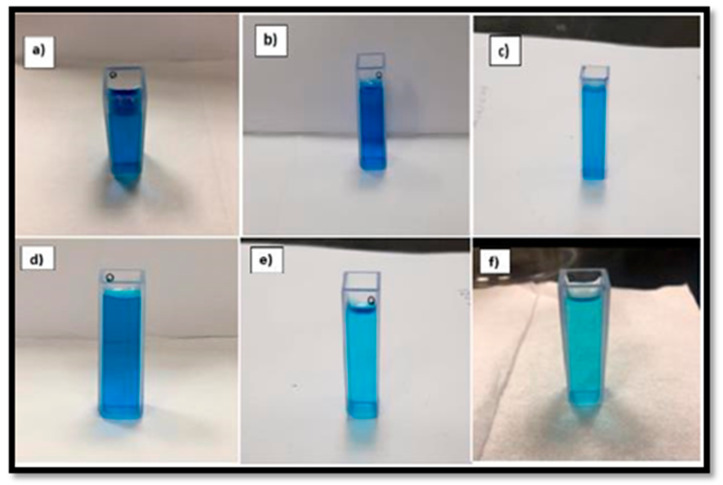
Degradation in the color of MB with ZnO catalyst at (**a**) 0 min, (**b**) 30 min, (**c**) 60 min, (**d**) 90 min, (**e**) 120 min, (**f**) 150 min.

**Figure 12:**
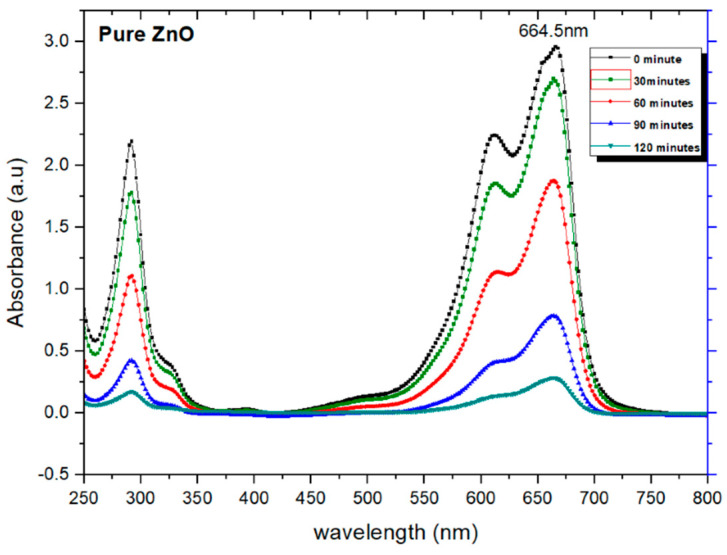
UV−vis absorption spectra for pure ZnO.

The possible mechanism for the degradation of the dye is demonstrated in Figure 10. Upon UV-vis light exposure, electrons (e−) and holes (h+) are created in the CB and VB on the surfaces of ZnO, respectively. The photo-created hot electrons (shifted from CB of ZnO to Au) react with the adsorbed O_2_ to produce O^2−^. These superoxide radicals react with water to generate H_2_O_2_, OH and *OH. These radicals (*OH, and O^2−^) and H_2_O_2_ are responsible for the degradation of organic dye molecules [12,54]. Herein, the Schottky junction between Au and ZnO builds an internal electric field that monitors the movement of electrons and holes in opposite ways. The surface plasma frequency of Au and the localized surface plasma resonance (LSPR) contribute to the visible light absorption process [55]. Figure 11 displays the photocatalytic degradation in the color of MB in the presence of ZnO catalyst from 0 to 150 min when the samples were collected at 30 min intervals. The gradual reduction in the darkness of the color of MB after each lap of exposure time has been noticed (Figure 11a–f). Figure 12 shows a gradual decrease in the absorption peak intensity around 280 nm and at 664 nm with an increase in irradiation time. Accordingly, UV-vis absorption spectra depict a photocatalytic degradation profile of MB for pure ZnO nanoparticles catalyst, as shown in Figure 12.

**Figure 13:**
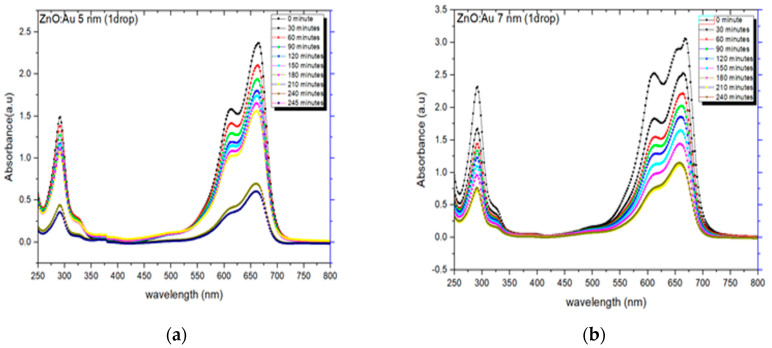

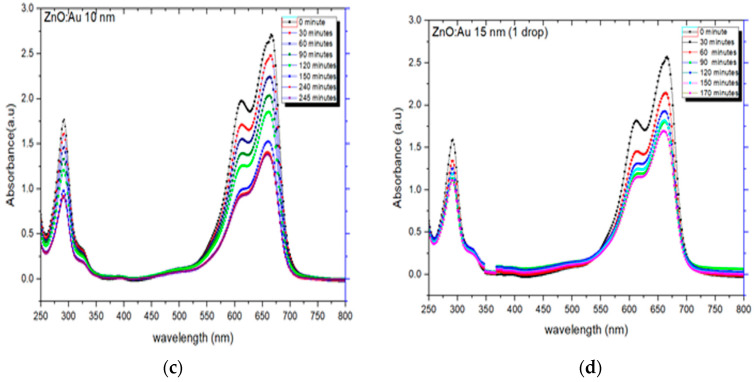
UV-vis absorption spectra depicting the degradation profile of MB in case of one drop (**a**) ZnO:−Au 5 nm, (**b**) ZnO:−Au 7 nm, (**c**) ZnO:−Au 10 nm, (**d**) ZnO:−Au 15 nm.

UV-vis absorption spectra exhibiting the photocatalytic degradation profile of MB for ZnO: Au-based photocatalyst prepared with Au NPs of size 5 nm, 7 nm, 10 nm, and 15 nm are shown in Figure 13. It can be observed from these spectra (Figure 13a–d) that there is a gradual decrease in the intensity of the MB absorption peaks at 280 nm and 664 nm, with an enhancement in the irradiation time. It can also be observed that despite more enhancement in the irradiation, even up to 240 min as compared to pure ZnO photocatalyst (irradiation time of 120 min), a significant decrease in the intensity of the absorption peaks is not noticed. Additionally, with ZnO: Au photocatalysts with Au nanoparticles size of 5 nm–15 nm, the intensity of the absorption peaks showed a gradual decreasing trend [12,31,43,56].

**Figure 14:**
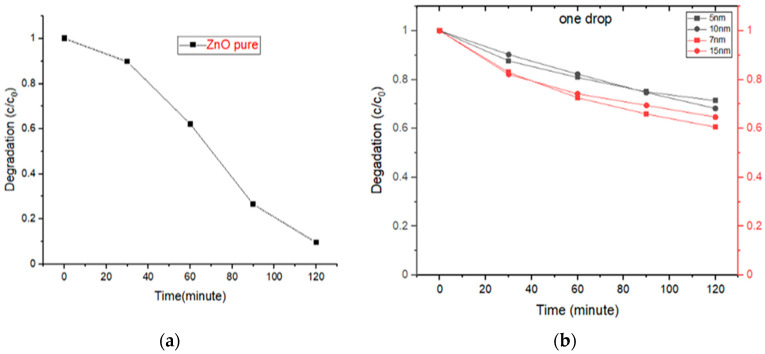
Plot of photodegradation vs. time for (**a**) pure ZnO and (**b**) Au-decorated ZnO thick films.

Figure 14a,b show the decolorization efficiency of pure ZnO and ZnO: Au-based photocatalysts regarding MB’s concentration change (C/C_0_) and the maximum absorbance values (A/A_0_), respectively. Here, C_0_ represents the initial concentration of the solution, and C represents the dye concentration at time t. The decolorization efficiency was proportional to maximum absorbance values (A/A_0_), resulting from the observed values of different concentrations for ZnO pure powder and ZnO: Au obtained from the UV spectrum and Table 5. However, the efficiency of pure ZnO photocatalyst is better than the ZnO: Au-based one. As observed in UV-visible spectra, this efficiency can be attributed to the decreased irradiation transmission due to masking by Au NPs.

It is inferred from Figure 14 that the photocatalytic activity increased with the increase in Au Nps size, although it is less than a pure ZnO-based photocatalyst [43].

## 3. Experimental Details

### 3.1. Materials

The citrate-based Biopure Gold (Au) nanoparticles of different sizes were purchased from Nano Composix San Diego, CA, USA. The materials used were as follows: 2-methoxy ethanol (C_3_H_8_O_2_) as a solvent [99.8%, Sigma Aldrich, St. Louis, MO, USA], zinc acetate dihydrate (Zn (CH_3_COO)_2_·2H_2_O, mw = 219.5 g/mol) [98%, BDH chemicals Limited, Poole Dorset, UK] as the precursor and potassium hydroxide (KOH)) as pH controlling agent [85% pellets, Sigma Aldrich]. Gold nanoparticle solutions used to sensitize ZnO films have particle sizes ranging from 5 nm, 7 nm, 10 nm, to 15 nm.

### 3.2. Preparation of ZnO Quantum Dots

**Figure 15:**
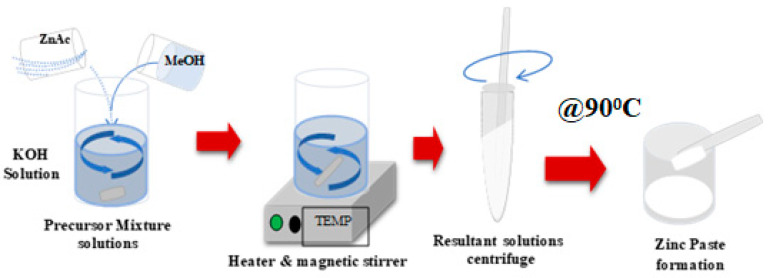
The schematic diagram of the resultant mixture of zinc thin film paste.

Using a typical ZnO quantum dots synthesis process, 23 mmol (0.631 g) of zinc acetate dihydrate (Zn (CH_3_COO)_2_·2H_2_O) was taken in a clean glass beaker (beaker ‘A’) [27,28]. Briefly, 125 mL of methanol (MeOH) was added to this beaker under magnetic stirring and the temperature was raised to 60 °C to dissolve zinc acetate completely. In another beaker (beaker ‘B’), a 1.57 g of KOH dissolved in 65 mL of MeOH was prepared under magnetic stirring. The solution from beaker’ B’ was added to beaker ‘A’ slowly and continued heating for 90 min when the reaction solution became turbid from transparent. The reaction mixture was still heated to 60 °C and stirring was continued for another 60 min until noticing that the mixture color has become milky. This resultant mixture was washed with methanol five times and centrifuged to get a white-colored semi-solid mass settled at the bottom of the centrifuge tube. Finally, this semi-solid mass was put on a clean glass Petri-dish and heated inside the oven for 60 min at 90 °C, as shown in Figure 15. The weight of the dried powder was measured to be 0.254 g.

### 3.3. Preparation of ZnO Thick Films by Dr. Blade Technique

**Figure 16:**
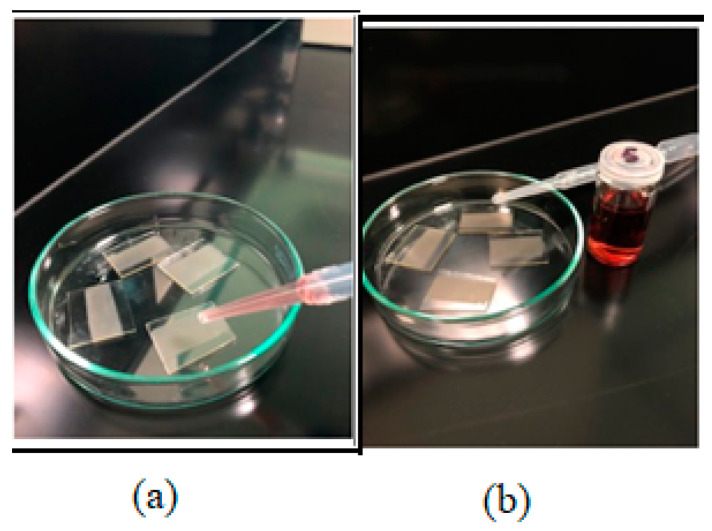
Typical Au-decorated ZnO thick films prepared via drop-casting with Au NPs solution: (**a**) before and (**b**) after Au drop-casting.

ZnO thick films were deposited on glass substrates using the doctor blade technique. First, 1 g of ethyl cellulose was dispersed in 9 mL of ethanol and kept for two days to get the honey-like viscous gel. In 2.26 g of this gel, 1.2 g of finely ground ZnO powder with 4.54 g of alpha-terpineol was added. Then, this semi-solid mass was mixed with a glass rod for a minute, followed by magnetic stirring for about 60 min. At the end of this process, ZnO paste was ready for use [28,29,30]. Each glass substrate was cut into three pieces with the diamond tip glass cutter to carry out the doctor blade process. Using a special cello-tape (which has very small thickness), we covered the edges/sides of the glass substrate, dropped a small amount of ZnO paste at one end, and then spread it with the clean glass rod on the glass substrate. Afterward, the attached cello tapes were taken off, and the glass substrates were kept in the furnace for 60 min at 450 °C to burn off the organic contents of the paste. Then, the furnace was allowed to cool down naturally overnight to get the final ZnO films deposited on a glass substrate, as shown in Figure 16.

### 3.4. Decoration of ZnO Films with Au via the Drop-Casting Process

The drop-casting method was used to sensitize ZnO films by Au nanoparticles. Typically, one drop of Au nanoparticles was deposited on the ZnO film of each substrate using the dropper, allowing it to spread all over the film and after two hours, it was put in the furnace at 90 °C for 10 min, and then allowed to cool down. The resultant films were used for further physico-chemical characterization (Figure 16).

### 3.5. Physico-Chemical Characterization of Au-Decorated ZnO Thick Films

The X-ray diffraction patterns for pure and Au-loaded ZnO films were obtained using a diffractometer (MRD System X’Pert D8 Advance, copper source with λ = 1.54060 Å, 45 kV, and 40 mA). The optical properties of the undoped and Au-doped ZnO films were studied using a UV-visible spectrophotometer (Spectro UV-VIS double beam P.C. 8 scanning auto cell (UVD-3200)-Biochrome. The morphological properties of undoped and Au-doped ZnO films were obtained using JEOL-JSM (7610 F) field emission scanning electron microscope (FESEM). The compositional analysis was also carried out using the Energy Dispersive X-ray Spectroscopy/Analysis (EDS/EDAX) system attached to the scanning electron microscope. The average particle size was calculated from FESEM images using the ImageJ program. The information on the particle size was also obtained using a transmission electron microscope (JEOL, Akishima, Japan, JEM-1400 at an operating voltage of 100 kV) after dispersing the scrapped-off portion of the film in ethanol, depositing a drop of this solution over a polymer-coated Cu grid and drying at room temperature.

### 3.6. Photocatalytic Dye Degradation Studies

The photocatalytic investigations using Lelesil innovative systems were performed under UV-vis light with a 300 W (Xenon lamp) to degrade MB (an organic dye). ZnO nanopowder (50 mg) was mixed with the MB stock solution as a catalyst. We prepared 100 mL of MB solution (1.598 mg of MB in 100 mL of distilled water) under magnetic stirring for 30 min in the dark to achieve an adsorption equilibrium of MB with the photocatalyst. Then, the mixture was exposed to UV-vis light at room temperature. Every 30 min, we took a small amount (for example, 5 mL; See Section 2.7) of it from the reactor and added the same amount of distilled water into the reactor, and carried out UV absorption reading (spectrum) of the solution until an overlap was noticed or the reading reached the end (See Section 2.7) [31].

## 4. Conclusions

Pure ZnO nanopowders were prepared using the chemical hydrolysis method. Thick films of the synthesized nanopowders were deposited on glass substrates using the doctor blade technique. Au decoration of the ZnO nanoparticles in the films was carried out via drop-casting Au NPs solution of sizes 5 nm, 7 nm, 10 nm, and 15 nm. X-ray diffraction study revealed that all the films crystallized to a hexagonal wurtzite structure. From optical studies, the highest value of Eg = 3.28 eV was found in undoped ZnO films, and with Au doping, the energy gap was found to be decreased, which substantiates the redshift. Photoluminescence spectra of pure and Au-decorated ZnO films exhibited exciton emission peaks in the ultraviolet (UV) region and blue and blue-green band emissions in the visible region. TEM studies revealed that the particle size of nanopowders is around 5–10 nm which falls in the range of QDs (quantum dots) that have a spherical shape. Finally, the photocatalytic activity of pure ZnO and ZnO: Au samples was investigated using MB (methylene blue) organic dye. The maximum absorbance decreased as the exposure time increased from 0 to 120 min which signifies the photodegradation of MB mixed with pure ZnO. The study on MB using ZnO: Au photocatalysts (with different Au particle sizes) revealed that degradation of MB occurred between 170 and 245 min. Substantial degradation efficacy of 90.2% methylene blue (MB) was achieved in natural pH in 120 min using pure ZnO catalyst while one drop gold-loaded catalysts, ZnO: Au 5 nm, ZnO: Au 7 nm, ZnO: Au 10 nm and ZnO: Au 15 nm, conveyed MB degradation efficacy of 74.5% (in 245 min), 63.8% (240 min), 49.6% (240 min) and 34.0% (170 min) in natural pH, respectively. This result implies that the photocatalytic dye degradation efficiency of pure ZnO is better than ZnO: Au samples.

## Figures and Tables

**Table 1:** The average crystallite size, lattice strain, and dislocation density from XRD (for one drop Au).

Au NP Size (nm)	Average Crystallite Size from XRD (nm)	Lattice Strain (%)	Dislocation Density (nm^−2^)
0 nm	30.56	5.78 × 10^−3^	0.001998596
5 nm	89.32	4.86 × 10^−3^	0.002493226
7 nm	54.37	3.76 × 10^−3^	0.000962403
10 nm	47.49	3.15 × 10^−3^	0.000535229
15 nm	112.88	3.40 × 10^−3^	0.000785233

**Table 2:** EDS compositional data.

	Pure ZnO		ZnO: Au (5 nm)		ZnO: Au (7 nm)		ZnO: Au (10 nm)		ZnO: Au (15 nm)	
	Weight%	Atomic%	Weight%	Atomic%	Weight%	Atomic%	Weight%	Atomic%	Weight%	Atomic%
Zn	60.89	27.59	70.25	35.99	67.35	34.36	70.50	37.09	72.22	30.34
O	39.11	72.41	30.65	64.16	31.38	65.42	29.25	62.87	27.21	60.56
Au	0	0	−0.91	−0.15	1.27	0.22	0.25	0.04	0.58	0.10
Total	100	100	100	100	100	100	100	100	100	100

**Table 3:** Average particle size data obtained from FESEM and TEM. Elemental composition data obtained from EDS.

Characterizations	Pure ZnO	ZnO: Au (5 nm)	ZnO: Au (7 nm)	ZnO: Au (10 nm)	ZnO: Au (15 nm)
FESEM average Particle size (nm)	34.39	63.13	62.22	71.48	76.17
HRTEM average Particle size (nm)	9.00	6.00	8.00	5.00	6.00

**Table 4:** Variation of ZnO bandgap concerning Au Nps decoration in ZnO thick films.

Au Concentration	Band Gap (eV)
0 nm	3.280
5 nm	3.245
7 nm	3.241
10 nm	3.238
15 nm	3.200

**Table 5:** The overlap time and degradation efficiency in the spectrum for one drop doping of the ZnO: Au catalyst.

S. No.	Catalyst with MB	Overlap Time Degradation in the Spectrum Efficiency (Minutes) (%)
1	Pure ZnO	120 90.2
2	ZnO: Au 5 nm	245 74.5
3	ZnO: Au 7 nm	240 63.8
4	ZnO: Au 10 nm	240 49.6
5	ZnO: Au 15 nm	170 34

## Data Availability

Data presented in this study are available and will be provided on request.

## Sample Availability

Samples of the compounds are not available from the authors currently.
